# Transient reduction in dendritic spine density in brain-specific profilin1 mutant mice is associated with behavioral deficits

**DOI:** 10.3389/fnmol.2022.952782

**Published:** 2022-08-03

**Authors:** A. Özge Sungur, Caroline Zeitouny, Lea Gabele, Isabell Metz, Markus Wöhr, Kristin Michaelsen-Preusse, Marco B. Rust

**Affiliations:** ^1^Molecular Neurobiology Group, Institute of Physiological Chemistry, University of Marburg, Marburg, Germany; ^2^Behavioral Neuroscience, Experimental and Biological Psychology, University of Marburg, Marburg, Germany; ^3^Department of Cellular Neurobiology, Technical University (TU) Braunschweig, Braunschweig, Germany; ^4^Deutsche Forschungsgemeinschaft (German Research Foundation) (DFG) Research Training Group, Membrane Plasticity in Tissue Development and Remodeling, Graduiertenkolleg (Gradeschool) (GRK) 2213, University of Marburg, Marburg, Germany; ^5^Social and Affective Neuroscience Research Group, Laboratory of Biological Psychology, Research Unit Brain and Cognition, Faculty of Psychology and Educational Sciences, Katholeike Universiteit (KU) Leuven, Leuven, Belgium; ^6^Leuven Brain Institute, Katholeike Universiteit (KU) Leuven, Leuven, Belgium; ^7^Center for Mind, Brain and Behavior (CMBB), University of Marburg and Justus-Liebig-University Giessen, Marburg, Germany

**Keywords:** actin dynamics, spine morphology, memory formation, social recognition, object recognition, ultrasonic vocalization

## Abstract

Actin filaments form the backbone of dendritic spines, the postsynaptic compartment of most excitatory synapses in the brain. Spine density changes affect brain function, and postsynaptic actin defects have been implicated in various neuropathies. It is mandatory to identify the actin regulators that control spine density. Based on previous studies, we hypothesized a role for the actin regulator profilin1 in spine formation. We report reduced hippocampal spine density in juvenile profilin1 mutant mice together with impairments in memory formation and reduced ultrasonic communication during active social behavior. Our results, therefore, underline a previously suggested function of profilin1 in controlling spine formation and behavior in juvenile mice.

## Introduction

Most excitatory synapses of the mammalian brain are formed on small dendritic protrusions termed dendritic spines, which play a major role in integrating synaptic input (Bosch and Hayashi, 2012). The morphologies of dendritic spines range from filipodia-like protrusion to mushroom-like structures, and their density as well as their size and morphology, can change in response to neuronal activity (Hering and Sheng, 2001). This specific form of structural plasticity contributes to neuronal excitation, and changes in dendritic spine density or morphology have been implicated in human brain disorders (Phillips and Pozzo-Miller, 2015; Spence and Soderling, 2015; Pelucchi et al., 2020a). Filamentous actin (F-actin) forms the structural backbone of dendritic spines (Bosch and Hayashi, 2012), and actin-binding proteins (ABP) that control the postsynaptic actin cytoskeleton gained increased attention as critical regulators of synapse physiology and brain function (Rust et al., 2010; Bosch et al., 2014; Spence and Soderling, 2015; Wolf et al., 2015; Zimmermann et al., 2015). Moreover, dysregulation of the postsynaptic actin cytoskeleton has been implicated in neuropsychiatric diseases such as autism spectrum disorders (ASD), schizophrenia, and intellectual disability, as well as in Alzheimer’s disease (Duffney et al., 2015; Spence and Soderling, 2015; Pelucchi et al., 2020b).

Profilins are structurally conserved proteins that are best known as accelerators of nucleotide exchange on globular actin monomers (G-actin) required for F-actin assembly (Witke, 2004; Rust et al., 2012; Murk et al., 2021). Two of the four profilins, namely profilin1 and profilin2, are expressed in the mouse brain (Witke et al., 1998), and both have been located in postsynaptic structures of isolated neurons and implicated in dendritic spine morphology (Ackermann and Matus, 2003; Neuhoff et al., 2005). Supported by enhanced postsynaptic profilin immunoreactivity that was accompanied by spine enlargement upon fear conditioning in rats (Lamprecht et al., 2006), these studies suggested important functions for profilin1 and profilin2 in dendritic spine morphology and structural plasticity. This view has been challenged by the analyses of gene-targeted mice lacking either profilin1 or profilin2 (Pilo Boyl et al., 2007; Görlich et al., 2012). However, more recent studies in organotypic slice cultures and primary hippocampal neurons confirmed a role for profilin2 in spine morphology and structural plasticity, and they suggested that profilin1 was rather relevant for spine formation (Michaelsen et al., 2010; Michaelsen-Preusse et al., 2016). By exploiting brain-specific knockout (KO) mice, we here tested the hypothesis that profilin1 is relevant for spine formation and behavior in juvenile mice.

## Materials and methods

### Mice

Generation of brain-specific profilin1 KO mice (Pfn1*^flx/flx,Nes–Cre^*) has been reported earlier (Kullmann et al., 2012a). Pfn1*^flx/flx^* littermates were used as controls (CTR). Mice were housed in the animal facility of the University of Marburg on 12-h dark–light cycles with food and water available *ad libitum*. Treatment of mice was in accordance with the German law for conducting animal experiments and followed the guidelines for the care and use of laboratory animals of the U.S. National Institutes of Health. Behavioral experiments were approved by the Regierungspräsidium Gießen (reference: V54-19c2015h01MR20/30 Nr. G40/2016) and the killing of mice for organ removal by local authorities (references: AK-7-2014-Rust, AK-11-2020-Rust).

### Spine analysis

FD Rapid GolgiStain™ kit (FD Neurotechnologies, Columbia, MD, United States) was used for Golgi-Cox staining. Tissue impregnation and tissue section staining were performed according to the manufacturer’s instructions. Transversal sections of 150 μm thickness were cut with a vibrating microtome (VT1200S, Leica, Wetzlar, Germany) while embedded in 2% agar in 0.1 M PBS. Each section was mounted on an adhesive microscope slide pre-coated with 1% gelatin/0.1% chromalaun on both sides and stained according to the manufacturer’s protocol with the exception that AppliClear (Applichem, Darmstadt, Germany) was used instead of xylene. Finally, slices were mounted with Permount™ (Thermofisher Scientifics, Waltham, MA, United States).

For analysis of spine density imaging of second or third order branches of apical dendrites of CA1 pyramidal neurons was performed (z-stack thickness of 0.5 μm) using an Axioplan 2 imaging microscope (Zeiss, Oberkochen, Germany) equipped with a 63x (N.A. 1.4) oil objective and a digital camera (AxioCam MRm, Zeiss, Oberkochen, Germany). The number of spines was determined per micrometer of dendritic length using the ImageJ software (1.48v, National Instruments of Health, United States). Spine density of 4–5 dendrites (continuous dendrite stretches of 50–100 μm) was averaged per animal. Data were analyzed using Graphpad Prism (Version 7) software. Spine density is expressed as mean values (MV) ± standard error of the means (SEM). To analyze the spine type numbers of apical dendrites in the CA1 region, images were further processed with ImageJ (1.53c, National Instruments of Health, United States). The filter “gaussian blur” (radius 25) was applied to the image stack and subtracted from the same image stack processed with the “unsharp mask” filter (radius 5, mask weight 0.6) by the use of the “image calculator” of ImageJ. After image processing, the diameter, as well as the length of each spine, was measured manually. For spine type assessment, the following criteria were used: head diameter < 0.6 μm (mushroom), spine length > 2 μm (filopodia), head diameter/spine length ≤ 1 (stubby), 1 μm > spine length < 2 μm AND head diameter < 0.6 μm (long thin), spine length < 1 μm AND head diameter < 0.6 μm (thin). Data are presented as mean values ± standard errors of the mean (SEM). A *p*-value of < 0.05 was considered significant. Data were analyzed using 1- and 2-way ANOVA, details are reported in the text.

Reciprocal social interaction was tested between postnatal days (P) 23–27. Social approach, social recognition, and novel object recognition were performed between P25 and P30. To measure reciprocal social interaction, pairs of juvenile mice were allowed to socially interact for 5 min after one mouse of the pair was habituated to the test environment for 1 min, using a previously established protocol (Wohr et al., 2015). Only same-sex/same-genotype pairs consisting of non-littermates were used. To enhance the level of social motivation, juvenile mice were socially isolated for 24 h before testing. Testing was performed in a clean Makrolon type III cage with fresh bedding and a metal lid under dim red light. Behavior was recorded using a video camera placed 30 cm away from the cage.

Social approach, social recognition, and novel object recognition were performed in a three-chambered box, similar to our previous studies (Sungur et al., 2017). The box was made of dark gray polycarbonate material and consisted of two side chambers (230 × 345 × 350 mm) connected through a smaller chamber (145 × 70 × 350 mm) located centrally between both side chambers. This middle chamber had two retractable doors to control access to the side chambers. Behavioral testing in the three-chambered box was conducted on three consecutive days. On the first day, subject mice were individually kept for 30 min in a Makrolon type III cage and were then allowed to explore the empty three-chambered box for 30 min to habituate to the apparatus. On the second and third days, subject mice were again placed in this chamber for 30 min. Subsequently, social behavior paradigms or non-social memory tasks were performed in balanced order, with social approach and social recognition being performed on 1 day and novel object recognition on the other day.

#### Social approach and social recognition

After being individually kept in a Makrolon type III cage for 30 min, subject mice were tested for social approach and social recognition (Nadler et al., 2004), using a modified protocol previously established (Sungur et al., 2017). Testing consisted of three phases, that is, social approach trial (10 min), inter-trial interval (30 min), and social recognition trial (10 min). In the social approach trial, subject mice were allowed to freely explore for 10 min the three-chambered box containing an empty wired cage (object, non-social stimulus) in one side chamber and a stimulus mouse (age- and sex-matched wildtype mice) constrained in an identical wired-cage (animal) in the other side chamber. The cylindrical-shaped wired cages (diameter: 10.5 cm, height: 11.8 cm) were constructed at the precision mechanics facilities of the Philipps-University Marburg. The cages had 2 mm thick metal bars spaced 7 mm apart and were closable with a lid. After the social approach trial, the subject mouse was individually kept for 30 min in the previously used Makrolon type III cage (inter-trial interval). Thereafter, subject mice were returned to the three-chambered box for a 10-min social recognition trial. During the social recognition trial, subject mice were given the choice between the stimulus mouse from the previous social approach trial (familiar mouse) in the side chamber where it was presented before or a novel stimulus mouse replacing the empty wired cage (novel mouse) in the other side chamber. As stimulus mice, age- and sex-matched C57BL/6N mice (Charles River Laboratories, NC, United States) were used. Stimulus mice were group-housed under similar conditions as subject mice and habituated to the wired cages for 30 min before testing. Location and stimulus mice presented were counter-balanced between subject mice.

#### Novel object recognition

After being individually kept for 30 min in a Makrolon type III cage, subject mice were tested for novel object recognition (Bevins and Besheer, 2006), using a modified protocol previously established (Sungur et al., 2017). This test consisted of three phases, that is, the object acquisition trial (10 min), the inter-trial interval (30 min), and the object recognition trial (10 min). During the object acquisition trial, subject mice were allowed to freely explore for 10 min the three-chambered box containing two identical sample objects, with one sample object being centrally placed in each of the two side chambers. Thereafter, the subject mouse was individually kept for 30 min in the previously used Makrolon type III cage (inter-trial interval). During that time, one of the objects from the object acquisition trial (familiar object) was replaced with a novel object of similar size but different color, shape, and material (novel object) to test object recognition memory. Specifically, one clean familiar object and one clean novel object were placed into the three-chambered box, where the two identical objects had been located during the object acquisition trial. After the 30 min delay, each subject mouse was returned to the three-chambered box for a 10-min object recognition trial and allowed to freely explore the familiar and the novel object. As objects, two silver iron cylinders (50 mm in diameter, 80 mm high) and two red metal cubes (50 × 50 × 80 mm) were used. The location and type of objects presented were counter-balanced between subject mice.

Ultrasonic vocalization (USV) emission during reciprocal social interaction was monitored by an UltraSoundGate Condenser CM 16 Microphone sensitive to frequencies of 15–180 kHz (flat frequency response between 25 and 140 kHz; ± 6 dB; Avisoft Bioacoustics, Berlin, Germany), placed 15 cm above the cage lid. The microphone was connected *via* an UltraSoundGate 416 USGH audio device (Avisoft Bioacoustics) to a personal computer, where acoustic data were recorded with a sampling rate of 250 kHz (16 bit) by Avisoft RECORDER (version 2.97). Interaction-induced USV was analyzed with Avisoft-SASLab Pro software (Version 5.2.05; Avisoft Bioacoustics). A fast Fourier transform was conducted (512 FFT length, frame size: 100%, Hamming Window and 75% time-window overlap), producing spectrograms at 488 Hz frequency resolution and 0.512 ms temporal resolution. Ultrasonic vocalizations were marked and counted by a trained observer blind to genotypes.

All behavioral tests were analyzed in videos by an experienced observer blind to the genotype using the Observer XT 10.0 software (Noldus Information Technology, Wageningen, Netherlands). For reciprocal social interaction, parameters of social behaviors included: facial sniffing (sniffing the nose and snout region of the partner), anogenital sniffing (sniffing the anogenital region of the partner), following (walking straight behind the partner, keeping pace with the one ahead), push past (squeezing between the wall and the partner), crawling under/over (pushing the head underneath the partner’s body or crawling over or under the partner’s body), social grooming (grooming the partner), and being socially inactive while having social contact (lying flat or standing still while maintaining close physical contact with the partner), according to previous studies (Terranova and Laviola, 2005; Yang et al., 2012; Wohr et al., 2015). All social behaviors were analyzed for frequency of occurrence (that is, number of bouts) and duration in 1 min time bins. In addition to social behaviors, non-social behaviors including rearing (number of times an animal reared on its hind legs), grooming (number of bouts of face, body, and genital grooming movements), and digging (number of bouts of digging in the bedding, pushing, and kicking it around) were counted. For novel object recognition, social approach and social recognition, number of entries into the chambers, the time spent therein, and object investigation were scored. Novel object recognition and social recognition were defined as spending significantly more time sniffing the novel than the familiar object or mouse, respectively (for details: (Sungur et al., 2017).

#### Statistical analysis of behavioral data

For the analysis of direct reciprocal social interaction and the concomitant emission of interaction-induced ultrasonic vocalization (USV), an ANOVA for repeated measurements with the between-subject factor genotype and the within-subject factor test duration was calculated. Novel object recognition, social approach, and social recognition were analyzed using paired t-tests for comparing stimuli within genotypes. For novel object recognition and social recognition, behavior recorded in the first 5 min of each trial was included in the statistical analysis, since habituation to novel stimuli is likely to occur in testing periods exceeding 5 min (Bevins and Besheer, 2006). For novel object recognition, in total, eight animals were excluded from the final analysis due to inadequate object exploration or to counterbalance the object type (due to some litters being bigger than others). Data are presented as mean values ± standard errors of the mean (SEM). A *p*-value of < 0.05 was considered significant.

## Results

**FIGURE 1:**
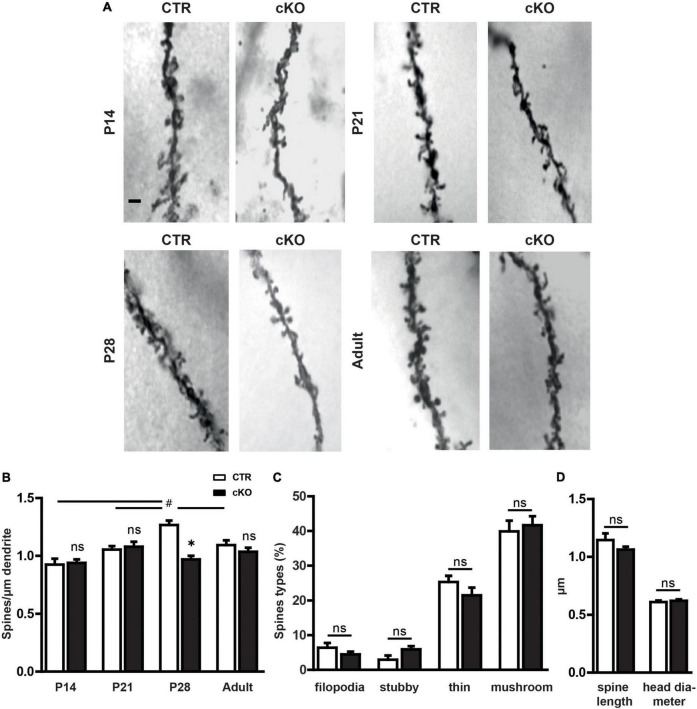
Profilin1 is relevant for dendritic spine density during a specific period of postnatal development. **(A)** Representative micrographs of CA1 apical dendrites in Golgi-stained brain sections from CTR and cKO mice at different developmental stages. Scale bar: 1 μm. **(B)** Quantification of total dendritic spine density at various postnatal stages; (spines/μm) P14: CTR: 0.92 ± 0.05, *n* = 3, cKO: 0.94 ± 0.03, *n* = 4, *p* > 0.99; P21: CTR: 1.05 ± 0.03, *n* = 4, cKO: 1.08 ± 0.04, *n* = 4, *p* > 0.99; p28: CTR: 1.30 ± 0.02, *n* = 4, cKO: 1.00 ± 0.03, *n* = 4, *P* < 0.05; adult: CTR: 1.10 ± 0.03, *n* = 4; cKO: 1.05 ± 0.04, *n* = 4. **(C)** Density of filopodia-like, thin, stubby, and mushroom-like spines in CTR and cKO mice at P28; (%) filopodia: CTR: 6.4 ± 1.4, cKO: 4.5 ± 0.8, *p* = 0.27; stubby: CTR: 2.9 ± 1.2, cKO: 6.0 ± 0.9, *p* = 0.09; thin: CTR: 25.4 ± 1.7, cKO: 21.5 ± 2.2, *p* = 0.22; mushroom: CTR: 39.0 ± 3.1, cKO: 41.7 ± 2.6, *p* = 0.67). **(D)** Dendritic spine length and head diameter in P28 CTR and cKO mice; length (μm) CTR: 1.15 ± 0.06, cKO: 1.06 ± 0.03, *p* = 0.23; diameter (μm) CTR: 0.61 ± 0.01, cKO: 0.62 ± 0.01, *p* = 0.6; 2-way ANOVA with Bonferroni *post hoc* test comparing both genotypes *p < 0.05, 1-way ANOVA with Tukey *post hoc* test within each genotype to reveal developmental alterations #*p* < 0.05 **(B)**. ^#^ Is part of Graph B p28.

Based on previous observations, we hypothesized a role for profilin1 in spine formation in the mouse brain. To test this hypothesis, we determined dendritic spine density in the hippocampal CA1 *stratum radiatum* from brain-specific profilin1 knockout (KO) mice during postnatal development. Generation of these mutants has been achieved by crossing a conditional strain (Pfn1*^flx/flx^*) and Nestin-cre (*^Nes–cre^*) transgenic mice (Tronche et al., 1999; Böttcher et al., 2009), and brain-specific profilin1 inactivation in these mice (termed cKO) has been validated before (Kullmann et al., 2012a). To determine spine density during postnatal development, we performed Golgi-Cox staining on brains dissected between P14 and P28 as well as in adult mice (Figure 1A). As we did not detect gender-specific differences, data from male and female mice were combined. Statistical analysis using a 2-way ANOVA revealed significant differences for factor age and genotype, as well as the interaction of both [*F*-age_(3,23)_ = 8.4, *p* < 0.001; *F*-genotype_(1,23)_ = 8.74, *p* < 0.01; *F*-interaction_(3,23)_ = 8.22, *p* < 0.001]. A Bonferroni *post hoc* analysis comparing genotypes directly at different ages revealed that there were no differences in total spine density between Pfn1*^flx/flx^* mice, which served as controls (CTR), and cKO mice at P14 and P21 (Figure 1B). Instead, total spine density was significantly reduced by 30% in P28 cKO mice. The phenotype was transient as spine density in adult cKO animals was not reduced (Figure 1B). To reveal differences in spine density during development, we performed a one-way ANOVA for each genotype separately. Significant differences in spine density during development were detected in CTR mice [*F*_(3,14)_ = 11.8, *p* < 0.001], which showed increased spine density at P28 when compared to P14 and P21 (P14: *p* < 0.001, P21: *p* < 0.05). P28 represented the peak in spine density as spine number in adult CTR mice was significantly lower when compared to P28 (*p* < 0.05). Notably, these developmental alterations in spine number were not observed in cKO mice as spine density did not differ significantly over time.

Next, we categorized dendritic spines according to their morphologies to test whether the defect in spine formation in P28 cKO mice might be accompanied by an impairment in spine maturation represented by an overabundance of filopodia or thin spines. Therefore, similar to previous studies (Hering and Sheng, 2001), we categorized dendritic spines as filopodia-like, thin, stubby, or mushroom-like spines and found no differences in the fractions of these spine categories between CTR and cKO mice (Figure 1C). In line with this, spine length, as well as head diameter, were not different between CTR and cKO mice (Figure 1D). Together, spine density was reduced in P28 cKO mice, but not at earlier stages or in adult mice, and reduced spine density was not accompanied by any alterations in the spine type distribution.

**FIGURE 2:**
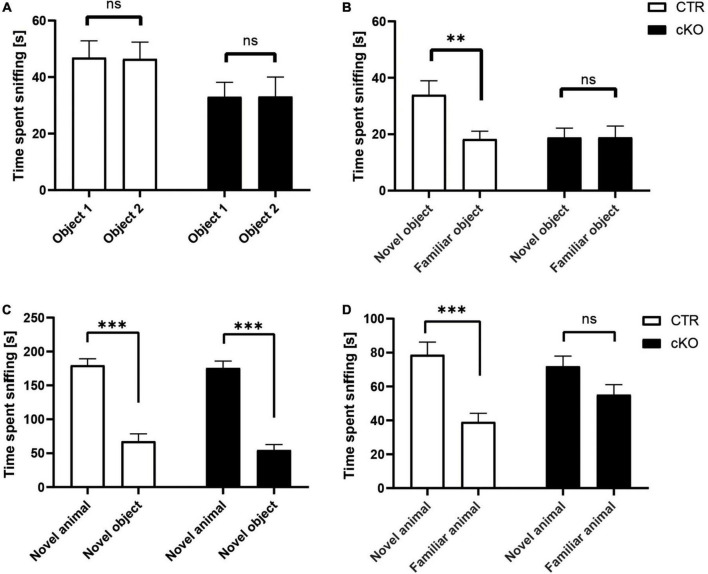
Normal social approach, but reduced social and object recognition in juvenile cKO mice. Graphs showing **(A)** time spent sniffing two identical objects (s), CTR: object 1: 46.9 ± 5.9, object 2: 46.4 ± 5.9, t_31_ = 0.135, *p* = 0.893, *n* = 32; cKO: object 1: 33.0 ± 5.1, object 2: 33.1 ± 6.9, t_35_ = 0.018, *p* = 0.986, *n* = 36. **(B)** Time spent sniffing novel and familiar object (s), CTR: novel: 34.0 ± 5.0, familiar: 18.3 ± 2.8, t_31_ = 3.226, *p* < 0.01; cKO: novel: 18.8 ± 3.3, familiar: 18.9 ± 5.3, t_35_ = 0.008, *p* = 0.994. **(C)** Time spent sniffing social and non-social stimulus (s), CTR: social: 179.7 ± 12.2, non-social: 67.6 ± 11.1, t_37_ = 5.854, *p* < 0.001; cKO: social: 175.7 ± 15.7, non-social: 54.8 ± 8.2, t_37_ = 6.673, *p* < 0.001. **(D)** Time spent sniffing novel and familiar social stimulus (s), CTR: novel: 78.7 ± 8.6, familiar: 39.1 ± 5.1, t_37_ = 3.983, *p* < 0.001; cKO: novel: 72.0 ± 8.1, familiar: 55.1 ± 8.1, t_37_ = 1.280, *p* = 0.209. ns: *p* = 0.05, **p < 0.01, ***p < 0.001.

The reduction in hippocampal spine density led us to investigate whether alterations in connectivity might be accompanied by cognitive impairments in juvenile animals as this brain region is crucially involved in memory formation (Martin and Clark, 2007). For this purpose, we performed the novel object recognition paradigm. During object acquisition, mice were allowed to explore two identical objects, and we recorded the time mice spent sniffing the objects (Sungur et al., 2018). As expected, CTR mice similarly explored both objects (Figure 2A). Likewise, cKO mice spent equal time sniffing the objects in both chambers. These data excluded any side preferences, which may impede data interpretation. In the test trial, we replaced one object with a novel object and again quantified the time mice were exploring both objects. CTR mice preferred the novel object, and they spent more time sniffing the novel than the familiar object (Figure 2B). Instead, cKO mice failed to discriminate between novel and familiar objects, and they spent equal time exploring both objects. Hence, juvenile cKO mice were unable to discriminate between novel and familiar objects during the object recognition trial, thereby demonstrating impaired object memory in these mutant mice. Besides its role in flexible cognitive function, the hippocampus is also connected to brain areas involved in social interactions such as the prefrontal cortex, amygdala, and cingulate (Rubin et al., 2014). Thus, we were also interested in potential impairments in social behavior in juvenile animals. Social approach, as well as social recognition, were tested in a three-chambered box, similar to our previous study (Sungur et al., 2018). To assess the social approach, we tested whether mice preferred a social stimulus (age- and sex-matched wildtype mouse) to a non-social stimulus (empty cage). As expected, CTR mice showed a strong preference for the social stimulus, and they spent much more time sniffing the cage with the social stimulus than the empty cage (Figure 2C). Similarly, cKO mice showed a strong preference for the social stimulus. Moreover, time exploring the social stimulus was not different between CTR and cKO mice (*t*_74_ = 0.202, *p* = 0.841). Hence, the social approach was normal in juvenile cKO mice. Social recognition was tested with a delay of 30 min. During social recognition, the previous stimulus mouse remained in the cage (familiar), while a novel stimulus mouse was placed in the previously empty cage, and we tested whether the subject mice showed a preference for the familiar or the novel social stimulus. As expected, CTR mice showed a preference for the novel social stimulus, as they spent more time sniffing the cage with the novel mouse (Figure 2D). In contrast, cKO mice showed no preference for the novel nor the familiar social stimulus. Of note, they spent almost similar time as CTR mice sniffing the novel social stimulus (*t*_74_ = 0.568, *p* = 0.572), while they spent more time sniffing the familiar social stimulus (*t*_74_ = 1.674, *p* = 0.098). Together, our data revealed impairments in object and social memory formation in juvenile cKO mice.

**FIGURE 3:**
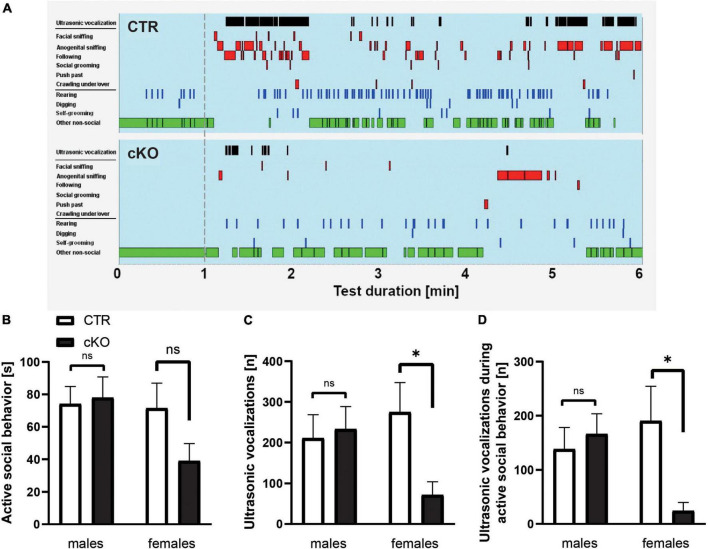
Normal reciprocal social interaction, but reduced ultrasonic vocalizations in juvenile cKO mice. **(A)** Representative ethograms of female CTR and KO pairs during a 5-min test phase (1–6, min right to the dashed line) upon 1 min of habituation (left to the dashed line). Depicted are ultrasonic vocalizations (black bars in row 1), six different social activities (i.e., facial sniffing, anogenital sniffing, following, social grooming, push past, and crawling under/over, red bars in rows 2–7), three different non-social activities (i.e., rearing, digging, and self-grooming, blue bars in rows 8–10), and other non-social activities (green bars in row 11). **(B)** Time spent in active social behavior (s), male CTR: 74.14 ± 10.7, cKO: 78.0 ± 12.8, F_1,19_ = 0.05, *p* = 0.825, *n* = 20 pairs; female CTR: 71.5 ± 15.5, cKO: 39.0 ± 10.7, F_1,15_ = 2.630, *p* = 0.127, *n* = 16 pairs. **(C)** Number of ultrasonic vocalizations (n), male CTR: 210.9 ± 57.6, cKO: 233.1 ± 55.5, F_1,19_ = 0.076, *p* = 0.786; female CTR: 274.7 ± 72.9, cKO: 71.4 ± 32.7, F_1,15_ = 5.328, *p* < 0.05. **(D)** Number of ultrasonic vocalizations during active social behavior (n), male CTR: 138.2 ± 40.0, cKO: 166.0 ± 37.6, F_1,19_ = 0.254, *p* = 0.620; female CTR: 190.4 ± 63.9, cKO: 24.3 ± 15.6, F_1,15_ = 5.004, *p* < 0.05. ns: *p* = 0.05, *p < 0.05.

As the last step, we aimed to further characterize social impairments in cKO mice. To do so, we first quantified direct reciprocal social interaction in same-sex/same-genotype pairs (no littermates) by determining the time mice spent in active social behavior, similar to our previous studies (Wohr et al., 2015; Sungur et al., 2018). Total time engaged in active social behavior (facial sniffing, anogenital sniffing, following, social grooming, push past, and crawling under/over) was similar in male cKO and CTR mice, but more than 40% lower in female cKO mice when compared to female CTR mice (Figures 3A,B). However, this reduction did not reach statistical significance [*F*_(1,15)_ = 0.127]. Instead, the number of ultrasonic vocalizations (USV) was affected. USV is mostly emitted during active social behaviors in mice and is a prominent aspect of their social behavior repertoire (Sungur et al., 2018; de Chaumont et al., 2021). They serve important communicative functions and are typically altered in mouse models for neurodevelopmental disorders (Caruso et al., 2020). In female cKO mice, USV emission rates were reduced by roughly 75% during the 5 min direct reciprocal social interaction test period, and it was significantly different between groups (Figure 3C), while male cKO and CTR mice did not differ from each other. Moreover, a detailed temporal analysis revealed that there was a significant genotype by sex interaction for USV emitted during active social behavior [*F*_(1,35)_ = 4.596, *P* < 0.05]. The majority of USV occurred during an active social behavior in female CTR mice, but not in female cKO mice suggesting desynchronization of reciprocal social interaction from USV emissions. The number of USV events during active social behavior was reduced by 85% in female cKO mice (Figure 3D), whereas temporal analysis did not reveal any differences between male CTR and cKO pairs (Figure 3D). Together, juvenile female cKO mice displayed deficits in ultrasonic communication.

## Discussion

We here report the relevance of profilin1 for both hippocampal spine number and behavior in juvenile mice. Behavioral deficits in juvenile cKO were characterized by cognitive impairment and reduced USV during active social behavior. Profilin1 has been shown before to be involved in behavior during postnatal development, and previous mouse studies unraveled important functions for profilin1 in glial cell adhesion and radial migration of granule neurons during cerebellar development (Kullmann et al., 2012a,b, 2015), as well as in regulating the division mode and differentiation of neural progenitors in the neocortex (Kullmann et al., 2020). Reduced spine density in juvenile cKO is indeed in good agreement with an earlier study that implicated profilin1 in spine formation in cultured hippocampal neurons (Michaelsen-Preusse et al., 2016). Moreover, normal spine density in adult cKO mice confirmed an earlier study that reported unchanged spine density, spine morphology, and synaptic plasticity in the hippocampus of adult mutant mice lacking profilin1 specifically in excitatory synapses of the forebrain (Görlich et al., 2012). Whether the change in spine density in juvenile mice is causally linked to cognitive impairment and ultrasonic communication needs further investigation. Our findings strengthen the view that profilin1 is relevant for cellular processes during brain development, while profilin2 acquired specific functions in the adult brain (Pilo Boyl et al., 2007; Di Domenico et al., 2021). It needs to be determined in future if the reported reduction in spine number in the absence of profilin1 results from impairments in spine formation, stabilization, or spine elimination.

Interestingly, profilin1 acts downstream of the fragile X mental retardation protein state FMRP in neural progenitors (Saffary and Xie, 2011) and profilin1, as well as its Drosophila homolog chickadee, but not profilin2 have been implicated in the cellular defects of fragile X syndrome (FXS) pathology (Reeve et al., 2005; Michaelsen-Preusse et al., 2016). We recently showed that spine actin dynamics are impaired in the absence of profilin1 indicated by a strongly increased actin turn-over time (Michaelsen-Preusse et al., 2016). Moreover, a comparable phenotype could be observed in neurons derived from the FXS mouse model (Fmr1 KO), for which a reduction in profilin1 levels was shown. In line with this, overexpression of profilin1 rescued impaired spine actin dynamics back to baseline levels of control neurons (Scharkowski et al., 2018). This points to the crucial role of profilin1 in controlling spine actin polymerization rates. Reduced polymerization might indeed impair spine formation/stabilization processes. It is therefore tempting to speculate that profilin1 dysregulation contributes to the immature spine profile and behavioral deficits characteristic of FXS pathology, the most common monogenetic cause of ASD (Phillips and Pozzo-Miller, 2015; Richter and Zhao, 2021). ASD shows a remarkable overlap in behavioral symptoms with other neuropsychiatric disorders such as schizophrenia and intellectual disabilities: cognitive impairments are present in all three disorders, and social and communication deficits have been reported for ASD and schizophrenia (Fung et al., 2012; Millan et al., 2012; Volkmar and McPartland, 2014; Pasciuto et al., 2015). It has been therefore suggested that these pathologies share common mechanisms. Genotype differences seen in females but not males in our study might be due to females emitting more USV in general, in line with sex-dependent genotype differences seen in other mouse models for neurodevelopmental disorders (Beis et al., 2015).

Human genetic studies revealed enrichment of mutations in genes regulating F-actin in excitatory synapses for neuropsychiatric disorders (Ramakers, 2002; Gilman et al., 2011; Fromer et al., 2014), and some of the strongest candidate genes including FMR1 are known to be involved in F-actin regulation (Reeve et al., 2005; Durand et al., 2012; Steinecke et al., 2014; Duffney et al., 2015; Peykov et al., 2015). Dysregulation of profilin1 during postnatal brain development may hence not only contribute to FXS pathology but also the pathologies of other neuropsychiatric disorders.

## Data availability statement

The raw data supporting the conclusions of this article will be made available by the authors, without undue reservation.

## Ethics statement

The animal study was reviewed and approved by Regierungspräsidium Gießen (reference: V54- 19c2015h01MR20/30 Nr. G40/2016) (references: AK-7-2014-Rust, AK-11-2020-Rust).

## Author contributions

KM-P and MBR conceived and designed the research and wrote the manuscript. AÖS, CZ, LG, IM, and MW performed experiments. All authors analyzed data and approved the submitted version.
